# Asthma in pregnancy: An update

**DOI:** 10.1177/1753495X20965072

**Published:** 2020-11-01

**Authors:** Simon Couillard, Clare Connolly, Catherine Borg, Ian Pavord

**Affiliations:** 1 Respiratory Medicine Unit and Oxford Respiratory NIHR BRC, Nuffield Department of Medicine, University of Oxford, Oxford, UK; 2 Faculté de Médecine et des Sciences de la Santé, Université de Sherbrooke, Sherbrooke, QC, Canada

**Keywords:** Asthma, pregnancy, biomarkers, FeNO, blood eosinophils, maternal outcomes, fetal outcomes

## Abstract

**Aim:**

To update obstetric care providers about asthma management.

**Summary:**

Asthma is the most frequent comorbid chronic illness in pregnancy. Convincing evidence shows that uncontrolled asthma magnifies the risk of maternal, fetal and neonate complications. Unfortunately, one in four women take no inhaler during pregnancy, and it is likely that decreased adherence, rather than changes in pathology, explains uncontrolled maternal asthma. Patient surveys reveal a need for information and reassurance. Although some molecules are preferred in pregnancy, there is currently no basis to withhold any asthma medication – old or new. Biomarkers such as blood eosinophils and fractional exhaled nitric oxide are an effective way to assess the risk of asthma attacks and the likelihood of responding to inhaled steroids. Furthermore, practice-changing trials in mild asthma show that switching reliever-only regimens to as-needed ‘controller-and-reliever’ therapy is effective. We suggest that applying these changes can alleviate women’s concerns and improve outcomes.

## Introduction

Asthma is a disorder of the airways that is characterised by typical symptoms and attacks of severe bronchoconstriction arising from a complex interplay between chronic inflammation and disordered airway function. It is the most common chronic disease in pregnancy, affecting 3–12% of women.^1,2^ There is now a compelling evidence that poor asthma control and asthma attacks in pregnancy are associated with adverse pregnancy outcomes.^3^ Pregnancy should therefore prompt a review of management in order to achieve control and reduce the risk of acute asthma attacks. This article aims to update obstetric care providers on the stratification and management of pregnant women with asthma.

## Applied respiratory pathophysiology in pregnancy

### Healthy pregnancy and the airways

Healthy pregnancy brings clinically significant changes to the respiratory system that may impact the evaluation and management of asthma (Table 1).

**Table 1.** Selected changes in respiratory physiology in pregnancy.

Parameter	Change	Clinical correlate
Minute ventilation	↑↑ ad 50%	Subjective shortness of breath, mild respiratory alkalosis^a^
Tidal volume	↑↑ ad 40%	
Respiratory rate	= / ↑ <10%	
PaO_2_	= / ↑	Hypoxia abnormal^a^
FEV_1_	No change	Airflow limitation not explained by pregnancy – suspect airways disease
FVC	No change	
Flow-volume loop	No change	
Pulmonary resistance	=/↓ (unclear)	
Diaphragm height	4 cm elevation	Contributes to shortness of breath at term
TLC, ERV, RV and FRC	↓ / ↓↓	
Upper airway vascular and mucus congestion	↑	Pregnancy rhinitis (non-allergic), snoring

^a^At term, normal arterial blood gas values: pH 7.44, PaCO_2_ 30 mmHg, HCO_3_^–^ 30 mmol/L and PaO_2_ 105 mmHg.

FEV_1_: forced expiratory volume in 1 s; FRC: functional residual capacity; FVC: forced vital capacity; ERV: expiratory reserve volume; Pa: partial pressure in artery; RV: residual volume; TLC: total lung capacity.

Adapted from Magriples and Cpoel^4^ and Gaiser.^5^

**Figure 1.**
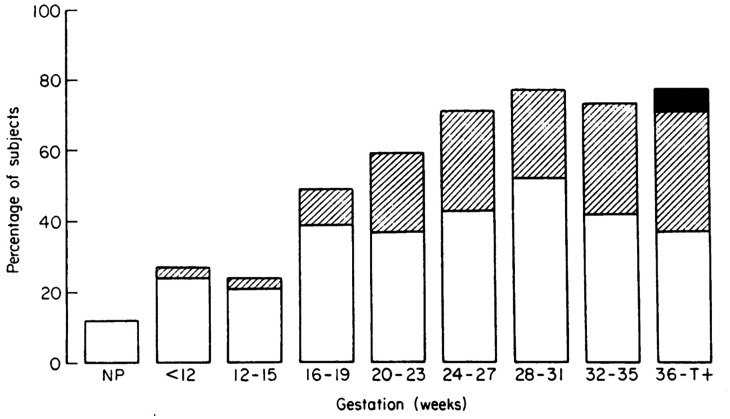
Incidence and severity of physiological dyspnoea during pregnancy. □: dyspnoea climbing more than one flight of stairs. ▪: dyspnoea while walking at an even pace on level ground, ■: dyspnoea on slightest exertion or at rest. Figure reproduced and legend adapted from Milne.^6^ NP: not pregnant.

Hormonal fluctuations alter the breathing pattern even in early pregnancy. Increased progesterone levels stimulate the respiratory centres in the brain, leading to an increase in minute ventilation, respiratory alkalosis and the ‘physiological dyspnea of pregnancy’, a sensation of shortness of breath reported by 25% of women in early pregnancy (Figure 1).^6^ Asthma-like symptoms can also occur as a result of progestin-induced relaxation of the lower oesophageal sphincter, heartburn and pharyngeal irritation.^7,8^ Pregnancy rhinitis is another confounding yet non-allergic, self-limited condition associated with increased placental growth hormone.^9–12^

Anatomic changes, although the most obvious manifestation of pregnancy and responsible for the changes in lung volumes noted in Table 1, have little consequence on the airways. However, the change in abdominal girth, pressure, diaphragmatic position and chest wall size contributes to the increasing incidence of physiological dyspnoea in later stages (Figure 1). Similarly, the enlarging uterus favours acid reflux. Nevertheless, there is no decrease in dynamic spirometry values.^13,14^ To be clear, airflow limitation (i.e. decreased FEV_1_ and an FEV1/FVC ratio <0.75) in pregnancy is nearly always abnormal (Table 1).

Immune changes in pregnancy are documented, yet poorly understood and of uncertain relevance to asthma. Reduced cell-mediated immunity probably explains the higher risk for infection from seasonal and/or H1N1 pandemic influenza.^15–18^ In mice, successful implantation requires a locally downregulated T-helper-1 type (Th)1 micro-environment – in favour of Th2 – to ensure the tolerance of the ‘fetal allograft’.^19–21^ Conversely, in humans, there is no predominance of circulating Th2 cytokines (i.e. interleukin (IL)-4, 5 and 13) or blood eosinophils in healthy pregnancy compared to non-pregnant women.^22^ In fact, plasmatic levels of eotaxin – an important eosinophil chemokine – decrease.^23^

To summarise, although shortness of breath, heartburn, rhinitis and viral infections can occur in normal pregnancy, lower airway pathology does not.

### Key pathological features of asthma

The fundamental features of asthma are chronic airway inflammation, structural changes to the airways and airway hyperresponsiveness.

#### Chronic airway inflammation

**Figure 2.**
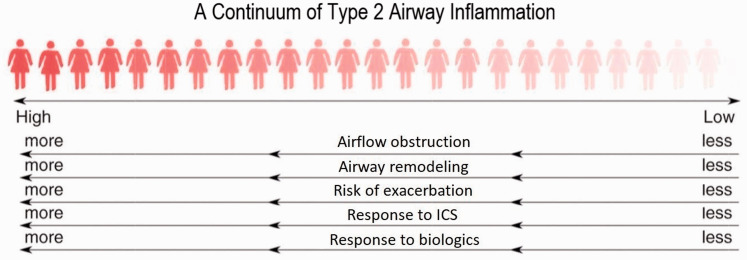
Type 2 driven inflammation is recognised as both a risk factor and a treatable trait. Figure and legend modified from Mason et al.^27^

The asthmatic airway inflammatory response is now acknowledged to be heterogeneous. In 40–70% of cases, it is characterised by varying degrees of airway eosinophilic infiltration orchestrated by type 2 cells, producing the key cytokines IL-4, 5 and 13.^24–26^ These cytokines increase production of allergen-specific immunoglobulin (Ig)E and play an important role in the maintenance of eosinophilic airway inflammation. Collectively, this response is known as type 2 airway inflammation (Figure 2).

Approximately half of women with asthma studied when stable and during an attack have no evidence of type 2 airway inflammation. This has been reported in severe asthma and in women with mild asthma who are not treated with inhaled corticosteroids (ICS), and the absence of eosinophilic airway inflammation has been confirmed by bronchoscopy studies.^28–30^ ‘Type 2-low’ asthma is associated with a lower risk of asthma attacks and a reduced response to corticosteroids.^31^

#### Structural changes to the airways

Structural changes in airway morphology (airway remodelling) occur as a result of chronic airway inflammation and dysfunction. Key features of airway remodelling include thickening of the sub-epithelial basement membrane caused by abnormal deposition of collagen; increased airway smooth muscle bulk; increased mucous-secreting cells and increased airway vascularity.^31^ It is thought that airway remodelling underlies the progressive airflow limitation seen in some women with asthma.

#### Airway hyperresponsiveness

Airway hyperresponsiveness represents an exaggerated bronchoconstrictor response to a variety of exogenous inhaled stimuli causing bronchoconstriction either by a direct effect on airway smooth muscle or by indirectly interacting with neural pathways or mast cells.^31^

### Pre-conception: Asthma and (sub)fertility

As it is frequently observed in chronic inflammatory diseases, women with asthma are more likely to be subfertile.^32–35^ Importantly, women treated with an ICS tend to have better fertility as opposed to no inhaler or short-acting beta-agonist (SABA)-only.^33,35^ There is still no high-quality prospective data to back ICS-use to optimise fertility in asthma. However, common sense and clinical experience suggest that uncontrolled and/or severe asthma hinder conception success.^36^

### Asthma and pregnancy

#### Obstetric risk varies with asthma control

**Figure 3.**
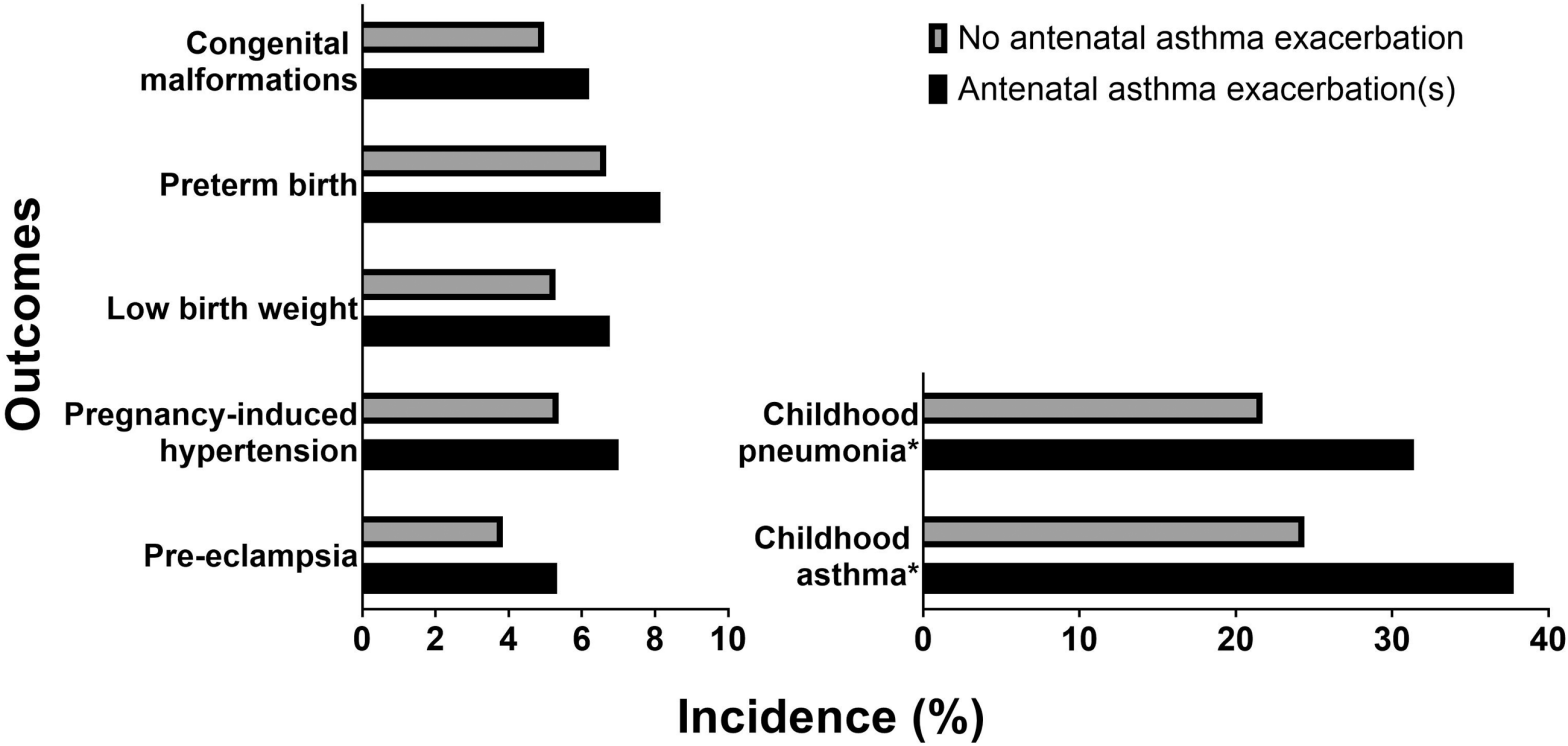
Incidence of adverse obstetric and perinatal outcomes in women with asthma who did and did not have asthma exacerbations during pregnancy. All outcomes listed were reported as significantly different after adjusted logistic regression analysis with generalized estimating equation for repeated measures or adjusted multivariable Poisson regression. Data from Abdullah et al.^3^ *Diagnosed before age 5.

Asthma is associated with a slight increase in maternal and fetal complications.^37–47^ Importantly, *uncontrolled* asthma magnifies these risks. A recent well-designed Canadian study of 103,424 singleton pregnancies in women with asthma showed that antepartum asthma attacks were associated with significantly higher rates of congenital malformations; preterm births; low birth weights, early life pneumonia and asthma; pregnancy-induced hypertension; and pre-eclampsia (Figure 3).^3^

#### Are the effects of pregnancy on asthma truly ‘unpredictable’?

Pregnancy often affects the course of asthma although this is variable and relatively unpredictable. Several studies have shown that asthma severity prior to conception,^48,49^ smoking,^50^ uncontrolled rhinitis^12^ and obesity^51^ are predictors of asthma attacks during pregnancy. The common wisdom that ‘one third of pregnant women experience an improvement in their asthma, one third experience a deterioration of their symptoms, and one third remain unchanged’ was confirmed by a meta-analysis of 14 studies.^52^ If symptoms do worsen, this is most likely in the second and third trimesters, with the peak incidence in month six.^53^ Peripartum and postpartum exacerbations are less frequent.^40^

Although many hypotheses exist, the mechanisms behind the aggravation of certain cases of asthma during pregnancy remain unclear.^54^ There is no tangible proof that type 2 airway inflammation – an important driver in asthma – increases spontaneously during pregnancy. Pregnancy-related conditions, micro-aspirations and susceptibility to viral infections may play a confounding role. Unfortunately, decreased adherence to asthma controller therapy seems the major factor at play.

#### The effect of pregnancy on adherence

The major role for poor treatment adherence is supported by pharmaco-epidemiological studies showing that asthma controller pick-up rates decrease by 17–30% during the first trimester.^55,56^ Prospective in-depth interviews reveal that lack of information, concerns about the safety of the medications and the desire for a ‘natural’ pregnancy were frequent reasons for discontinuation.^36,57^ Healthcare providers need to be able to answer the questions these women have about continuing asthma therapies and their safety throughout pregnancy and breastfeeding and, ideally, utilise strategies that ensure that the available treatments are used to their maximum potential.

## Evaluation

### Diagnosis

Characteristic clinical features (Table 2

**Table 2.** Clinical features that increase and decrease the probability that episodic respiratory symptoms are due to asthma.

Features that increase the probability of asthma
More than one of the following symptoms: cough, breathlessness, wheeze and chest tightness
Symptoms worse at night and in the early morning
Symptoms in response to exercise, allergen exposure and cold air
Symptoms after taking aspirin or b-blockers
History of atopic disorder
Family history of asthma and/or atopic disorder
Variable wheeze heard on auscultation of the chest
Variable PEF (see Table 3)
Otherwise unexplained low PEF
Features that lower the probability of asthma
Prominent dizziness, light-headedness and peripheral tingling
Isolated cough
Repeatedly normal physical examination of chest when symptomatic
Normal PEF when symptomatic
Voice disturbance
Symptoms with colds only
Chronic productive cough
Significant smoking history (i.e. >20 pack-years)
Cardiac disease

PEF: peak expiratory flow.

) coupled with objective demonstration of variable airflow obstruction (Table 3) and/or type 2 airway inflammation (Table 4) usually provide enough evidence to make the diagnosis of asthma.^58^ In uncomplicated pregnancies, spirometry is safe,^59^ but exercise and methacholine challenges are relatively contraindicated.^60,61^

**Table 3.** Confirming variable expiratory airflow limitation in adults.

Diagnostic feature	Criteria for making the diagnosis
Spirometry with reversible airflow obstruction	FEV_1_/FVC ratio decreased (<0.75–0.80) **AND** increase in FEV_1_ of >12% and >200 mL from baseline: 10 min after 400 mcg salbutamol **or** four weeks of anti-inflammatory therapy **or** between visits
Excessive variability in twice-daily PEF over two weeks	Average daily diurnal PEF variability > 10%
Positive exercise challenge^a^	Fall in FEV_1_ > 10% and >200 mL
Positive bronchial challenge^a^	Fall in FEV_1_ of 20% with methacholine concentration ≤8 mg/mL

^a^Avoid in pregnancy, as relatively contra-indicated.

FEV_1_: forced expiratory volume in 1 s; FVC: forced vital capacity; PEF: peak expiratory flow.

Adapted from Global Initiative for Asthma (GINA).^58^

**Table 4.** Biomarkers of type 2 airway inflammation.

Biomarker	Cut-off	Association with treatment response	Comments
Allergy testing	Variable	Anti-IgE	Increases probability of atopy, but does not confirm sensitisation; IgE levels do not predict clinical outcomes under omalizumab
Blood eosinophil count	≥150–300 cells/μL	CorticosteroidsAnti-IL-5Anti-IL4Rα	Generally available, cheap, associated with increased risk of asthma attacks
Sputum eosinophils	>2–3%	CorticosteroidsAnti-IL-5Anti-IL4Rα	Available at specialist centres, tissue specific, time-consuming
FeNO	>25–50 ppb	CorticosteroidsAnti-IL-4Rα	Quick, cheap, not specific; increases probability of asthma diagnosis; associated with increased risk of asthma attacks

FeNO: fractional exhaled nitric oxide; IgE: immunoglobulin E; IL: interleukin; R: receptor.

Adapted from Pavord et al.^62^

### Biomarker assessment

Biomarkers are useful surrogate measures of type 2 airway inflammation, the risk of asthma attacks and the likelihood that ICS will be effective (Table 4). An up-to-date full blood count (including eosinophils) and assessment of fractional exhaled nitric oxide (FeNO) are the most effective way of doing this. A blood eosinophil count <0.15 × 10^9^/L makes type 2 inflammation very unlikely to be present, whereas a count >0.3 × 10^9^/L makes it likely. The corresponding values for FeNO are 25 and 50 ppb. Blood eosinophils reflect IL-5 activity in the airway and systemically and FeNO IL-13 and 4 activity so these biomarkers likely have additive predictive and prognostic value. In keeping with this, type 2 inflammation and clinical outcomes associated with it are particularly likely if both biomarkers are elevated.^62^ There is now a convincing evidence that a treatment strategy that seeks to normalise FeNO results in better outcomes for mother and child than a symptom-guided approach.^63,64^

### Allergy testing

Confirming atopy with skin-prick testing or measurement of total IgE and allergen-specific IgE may help support a diagnosis of atopic asthma. Positive serology must be clinically correlated. We commonly check for sensitisation to *Aspergillus fumigatus*, house-dust mites and pollen; if there are any household pets, these are included. Testing can provide an important guide to allergen avoidance strategies and pharmacological treatments such as antihistamines, intranasal corticosteroids and, in certain cases, anti-IgE treatment.

## Management of asthma in pregnancy

### Aims

There are three major consequences of asthma and management goals that are directed at addressing each of these:

1. Control of asthma symptoms
2. Prevention of asthma attacks
3. Preservation of normal lung function

Importantly, these clinical outcomes involve different mechanistic pathways and therefore need different treatment approaches. Non-pharmacological and pharmacological measures are both important.

### Non-pharmacological interventions

Inadequate information, education and advice on managing asthma are key contributors to asthma exacerbations.

#### Patient education

Appropriate patient education is essential for the provision of patient-centred care. There are several aspects to this:

- Identifying and avoiding asthma triggers, most importantly smoking cessation;
- Understanding the role of different prescribed therapies (i.e. distinguishing ‘relievers’ and ‘controllers’);
- Encouraging compliance with medication and annual flu vaccination;
- Ensuring correct inhaler technique;
- Recording and monitoring peak flow;
- Following a self-management plan.

Specialist asthma nurses are central providers of information and education for women. Regular follow-up with an asthma nurse reinforces key messages and leads to superior asthma control.

#### Smoking cessation and allergen avoidance

Five pregnant women died from asthma in the UK between 2009 and 2015; all were current smokers.^65,66^ Smoking cessation improves asthma control, maternal health and fetal outcomes. Planning and reviewing pregnancy are ideal opportunities to start a cessation programme. Indeed, up to 75% of pregnant smokers successfully stop smoking by the end of pregnancy.^67–70^

Airborne allergen exposure and sensitisation are common and often contribute to increased asthma symptoms. Simple measures such as limiting contact with household pets, notably in the bedroom, should be counselled. Airborne and/or food allergen avoidance might be beneficial for the pregnant woman, but studies have not shown an effect on the risk of asthma in progeny.^71–73^

### Pharmacological therapy

Pharmacological therapies are central to asthma management. National, societal and international guidelines are very clear and consistent on the point that asthma treatment does not differ in pregnant women.^36,58,71^ The safety and effectiveness of continuing usual inhalers and asthma medications should be reinforced as early as possible. However, when introducing a new therapy before or during pregnancy, prescribers should choose molecules with the most re-assuring safety profiles.

#### Bronchodilators

Bronchodilators have no measurable effects on eosinophilic airway inflammation, and their use as first-line agents in women with asthma is no longer recommended. To be clear, even in mild asthma, there is evidence of harm from SABA-only treatment.^58^

*Beta-2 agonists* act by inhibiting contractility, leading to improvements in lung function and airway hyperresponsiveness. Following recent practice-changing trials in mild asthma,^74–77^ as-needed fast-onset long-acting β_2_-agonists in combination with an ICS (e.g. budesonide-formoterol or beclomethasone-formoterol) are the first step in asthma therapy. Since its authorisation for asthma in 2001, there here have been no reports of teratogenic or embryocidal effects of formoterol at usual doses (≤72 mcg/day). Of course, SABAs (e.g. salbutamol) can still be used to provide relief, but always in combination with a regular ICS.

Slower-onset LABAs include salmeterol, which has the longest track record of safety in pregnancy, and newer ‘ultra-long acting β_2_-agonists’ such as olodaterol or vilanterol. Although animal data suggest low risk for the latter two,^36^ these are not our first choice in pregnancy unless a once-daily LABA-ICS regimen is clearly required (e.g. fluticasone furoate and vilanterol once daily for adherence issues).

*Anti-muscarinic agents* cause bronchodilation by inhibiting vagal tone to the airways and have an additive effect to β_2_-agonists. Ipratropium is considered safe in pregnancy and is used in acute asthma attacks with minimal tachycardic effect on both mother and fetus.^78^ The long-acting anti-muscarinic (LAMA) tiotropium bromide has been observed to modestly decrease exacerbations in severe asthma. This class is especially useful in severe asthma with fixed airflow obstruction.^79^ Although experience in human pregnancy and lactation is minimal, LAMAs have been continued during pregnancy without concern.^78^

#### Corticosteroids

*ICSs* are the mainstay of asthma pharmacotherapy. Corticosteroids effectively suppress eosinophilic inflammation which is associated with marked improvement in symptoms, reduced exacerbation frequency and reduced asthma mortality. Response to this class of medication has been found to correlate with evidence of type 2 airway inflammation and, by extension, with blood eosinophil counts and FeNO levels. Importantly, ICSs reduce exposure to systemic steroids; the former administration route certainly has a much more reassuring safety profile than the latter.^78,80–97^ Budesonide, beclomethasone and fluticasone propionate are the preferred molecules for pregnancy. Noteworthy are the two randomised, placebo-controlled trials supporting the efficacy and safety of beclomethasone.^83,89^ There is no such data to support or to contraindicate the use of ciclesonide, mometasone or fluticasone furoate in pregnancy.

Women are often concerned about the possibility of adverse effects of ICS, and this belief is particularly prevalent in pregnant women. At low-to-moderate doses (budesonide ≤ 800 mcg/day, beclomethasone ≤ 400 mcg/day, fluticasone propionate ≤ 500 mcg), side effects are not significant. To minimise adverse effects, the use of spacer devices, dry powder mechanisms and mouth rinsing after inhaler use are counselled. At higher doses (lesser than the above doses), systemic absorption through the buccal and airway mucosa becomes increasingly important, and referral to a specialist is suggested.^58^

*Systemic corticosteroids* quickly suppress both airway and systemic eosinophilic inflammation in uncontrolled asthma.^98–101^ A short (five days) adequately dosed (e.g. 40 mg once daily) burst of oral prednisolone should never be withheld if clinically indicated for an acute exacerbation. Indeed, the benefits of its use greatly outweigh the potential areas of concerns this treatment raises.^36,71^

The risk of orofacial clefts is increased with systemic steroid use in conditions other than asthma and with maintenance doses.^102^ Pregnant women can be reassured that palatal closure is complete by the end of week 12, so teratogenic risk should be limited at further stages. Moreover, 90% prednisolone is metabolised by the placenta, with only 10% reaching the fetus.^71,103^ Finally, although preterm delivery, low birth weight and other adverse outcomes may be associated with exposure to systemic corticosteroids, one must consider confounding factors and a background rate of major birth defects in pregnancies of 2–4%.^104^

#### Anti-leukotrienes

Leukotrienes are important pro-inflammatory mediators that also promote bronchoconstriction. Cysteinyl-leukotriene receptor-1 antagonists (e.g. montelukast) have a modest suppressive effect in adults with asthma. They work best in women with exercise-induced symptoms, allergic rhinitis and/or aspirin-exacerbated respiratory disease. Although there have been worrying isolated case reports^105^ and low-quality retrospective publications,^106^ three well-designed studies have found no significant teratogenic effects.^107–109^ We have no qualms continuing this medication, but do not rely on montelukast to control asthma in adults and thus avoid its introduction in pregnant women.

#### Biologics

Biological agents (i.e. monoclonal antibodies) targeting IgE, IL-5, 4 and 13 have had a significant impact on clinical practice in severe asthma. Their exacerbation-preventing and corticosteroid-sparing effects are closely linked to easily measured biomarkers.^110^ Although all biological treatments are IgG-based and thus transported across the placenta in varying degrees according to gestational age and sub-type,^111^ there have been no concerns of teratogenicity despite being trialled and used in humans for more than two decades.

*Omalizumab* blocks the interaction of IgE with mast cells and basophils. IgE has an important effector role in allergic diseases, and suppression of IgE is therefore useful in the management of severe atopic asthma and/or urticaria. Clinical trials have shown fewer asthma attacks (∼25% decrease compared to placebo) and greater reductions in ICS doses with no apparent adverse effects.^58^ Evaluated in humans since 1995^112^ and marketed since 2003,^113^ omalizumab has a long track record of safety in pregnancy. A recent analysis of an exposure registration, prospective cohort of 250 pregnant women with asthma treated by this biologic showed no increase in adverse fetal outcomes when compared to the disease-matched external cohort.^92^ Omalizumab is generally preferred in women desiring children.

*IL-5 targeting agents* directly bind IL-5 (mepolizumab and reslizumab) or indirectly block its effect by binding to its receptor (benralizumab). This strategy has proven successful in severe eosinophilic asthma where they reduce asthma attacks (∼50% decrease), improve quality of life, allow withdrawal of oral corticosteroids (∼50% dose decrease) and slightly improve lung function. The benefits are greater in women with a high frequency of prior asthma attacks and in those with a higher blood eosinophil count.^58,114^ In pregnant non-human primates (NHP), administration of mepolizumab and benralizumab surrogate-antibody doses 9 and 310-fold, the maximum recommended human dose elicited no maternal or fetal adverse effect up to nine months after birth.^115,116^ There is no such data for reslizumab.^117^ In humans, the growing clinical experience, registries and publications for these biologics – mepolizumab has been trialled in humans since 2000^118^ – have not provided any signal of harm in pregnancy and breastfeeding.

*Dupilumab* inhibits IL-4 and IL-13 by binding to a common component of their receptors, the IL-4 receptor-alpha. This biological agent has a broad range of beneficial effects on asthma attack frequency (∼60% decrease), quality of life, lung function and oral corticosteroid dose reduction (∼50% decrease). Dupilumab is particularly attractive in women with comorbid conditions such as eczema^119^ and nasal polyposis^120^ as it is an effective treatment for both. The beneficial effects are closely related to blood eosinophil counts and FeNO levels. Experience in pregnancy is limited.^121^ In pregnant NHPs, doses 10 times the MRHD have been administered with no adverse outcome noted.^122^ In women of child-bearing age with severe uncontrolled type 2 high asthma, an appraisal of the benefit-risk ratio is key. We would rather initiate an ‘older’ biological molecule (i.e. omalizumab or mepolizumab) but have cautiously continued dupilumab in women that previously failed all other lines of therapy. Pharmacovigilance,^123^ spontaneous reporting of suspected adverse drug reactions^124^ and discussing registry enrolment are crucial in such cases.^122^

#### Other medications

- Methylxanthines (e.g. theophylline or aminophylline), although poorly tolerated, are safe throughout pregnancy and breastfeeding. More frequent dose-level monitoring is necessary due to decreased metabolism and protein-binding.^36^
- For allergic conditions, antihistamines (e.g. cetirizine or loratadine), intranasal corticosteroids (e.g. budesonide), skin emollients and mild-to-moderate topical corticosteroid creams (e.g. hydrocortisone 0.5–2.5%) are safe throughout pregnancy and breastfeeding.^119^
- Initiation of subcutaneous or sublingual immunotherapy during pregnancy is not recommended due to the possibility of severe allergic reactions. Women tolerating these therapies may cautiously continue if they derive clinical benefit.^125,126^

### Bringing it all together: State-of-the-art management of asthma in pregnancy

Guidelines recommend the titration of therapy for asthma in a stepwise manner, with the primary aim of satisfactorily controlling symptoms at the lowest dose of corticosteroid. Women with asthma should be reviewed pre-conception and more frequently until delivery. International guidelines recommend monthly assessments.^58^ This algorithm assumes clinical control and therefore fulfilment of all three targets of care. When there is concern that asthma control is suboptimal, consideration should be given to the changes in normal physiology – and adherence – that occur during pregnancy. Evaluation must be prompt and include objective measurements of uncontrolled asthma through spirometry and, considering recent evidence, biomarkers of type 2 airway inflammation (e.g. blood eosinophils and FeNO).

#### Biomarker-based management in pregnancy

In a landmark randomised-control trial of FeNO-based management of 220 non-smoking pregnant women with asthma, Powell et al.^63^ reported a striking reduction in moderate-to-severe exacerbations for the FeNO-and-clinical guided versus the clinical-only guided group (0.288 vs 0.615 exacerbations per pregnancy; 25 vs 41% women with at least one exacerbation). Although the trial was not powered to assess perinatal outcomes, there were favourable trends in the progeny of FeNO-managed mothers.

In 2019, a post-hoc analysis of this trial showed that the FeNO-guided management algorithm was equally fruitful in type 2 low asthma.^64^ Indeed, 103 (53%) of the women presented with a combination of low biomarkers of type 2 airway inflammation (FeNO < 30 ppb *and* blood eosinophils <260 cells/μ). In this ‘type 2-low group’, a lower median ICS dose combined with an increase in LABA therapy in the FeNO-guided arm was still associated with a decrease in exacerbations (19 vs 44% women with at least one exacerbation). Overall, treatment was better targeted to phenotype in the FeNO-guided algorithm. Earlier introduction of an LABA was observed in type 2 low asthma (11–30%) and ICS use increased in type 2 high asthma (48–86%). Biomarker-guided therapy during pregnancy is promising but will need further validation before being widely implemented.^127,128^

#### Management during labour

In labour, women with asthma should be offered the same options for pain relief as women without asthma. Prostaglandin E2 used in induction and oxytocin used for augmentation of labour can be used as normal. Peripartum and postpartum prescriptions should include continued use of inhalers.

In obstetric bleeding, prostaglandin F2α derivatives such as carboprost should be avoided as they can cause bronchoconstriction. Women who have taken more than 7.5 mg prednisolone daily for more than two weeks should be considered for parenteral hydrocortisone.^103^

## Conclusion

To summarise, asthma in pregnancy carries small risks yet great uncertainties for both the mother and her unborn child. We have shown compelling evidence that better control on maternal asthma favours better outcomes for both parties. Women need to be informed, reassured and empowered in their ability to control their airways disease through continued adherence to non-pharmacological and pharmacological advice. Hopefully, an up-to-date obstetric care provider can discuss newer and safer treatment regimens for their patient’s mild asthma such ICS-formoterol *pro rata necessitate*. In cases of uncontrolled and/or severe asthma, asthma specialists will be happy to provide advice in women appropriately diagnosed and stratified by biomarkers. A FeNO-guided algorithm enables precision medicine and results in reduced asthma attacks during pregnancy and improvements in pregnancy outcomes.
